# Unraveling the role of tomato Bcl-2-associated athanogene (BAG) proteins during abiotic stress response and fruit ripening

**DOI:** 10.1038/s41598-021-01185-7

**Published:** 2021-11-05

**Authors:** Mohammad Irfan, Pankaj Kumar, Irshad Ahmad, Asis Datta

**Affiliations:** 1 National Institute of Plant Genome Research, Aruna Asaf Ali Marg, New Delhi, 110067 India; 2 Plant Biology Section, School of Integrative Plant Science, Cornell University, Ithaca, NY USA; 3 Department of Biotechnology, Dr. Y.S. Parmar University of Horticulture and Forestry, Solan, Himachal Pradesh India

**Keywords:** Plant biotechnology, Plant molecular biology, Plant stress responses

## Abstract

B-cell lymphoma2 (Bcl-2)-associated athanogene (BAG) family proteins are evolutionary conserved across all eukaryotes. These proteins interact with HSP70/HSC70 and function as co-chaperones during stress response and developmental pathways. Compared to the animal counterpart, the BAG proteins in plants are much less studied and primarily *Arabidopsis* BAG proteins have been identified and characterized for their role in programmed cell death, homeostasis, growth and development, abiotic and biotic stress response. Here, we have identified BAG protein family (SlBAGs) in tomato, an economically important and a model fruit crop using genome-wide scanning. We have performed phylogenetic analysis, genes architecture assessment, chromosomal location and in silico promoter analysis. Our data suggest that *SlBAGs* show differential tissue specific expression pattern during plant development particularly fruit development and ripening. Furthermore, we reported that expression of *SlBAGs* is modulated during abiotic stresses and is regulated by stress hormones ABA and ethylene. In planta subcellular localization reveals their diverse subcellular localization, and many members are localized in nucleus and cytoplasm. Like previous reports, our protein–protein interaction network and yeast two-hybrid analysis uncover that SlBAGs interact with HSP70. The current study provides insights into role of SlBAGs in plant development particualry fruit ripening and abiotic stress response.

## Introduction

In the animal kingdom, B-cell lymphoma2 (Bcl-2) proteins are major regulators of apoptosis, the most studied form of programmed cell death (PCD)^1^. These proteins interact with Bcl2-associated athanogene (BAG) proteins which are evolutionary conserved across yeast, fungi, plants and animal kingdoms^2–4^. The members of BAG protein family function as co-chaperones that participate in diverse cellular functions including stress responses, proliferation, migration, and cell death^3,5,6^. The BAG protein was first identified in a search for Bcl-2-interacting proteins of a mouse embryonic cDNA library which enhances the anti-apoptotic effects of Bcl2^7^. Further, six BAG family members have been identified in humans which are involved in various cellular functions and physiological processes such as apoptosis, stress response, cell cycle regulation, carcinogenesis and neuronal differentiation^8^. BAG proteins are distinguished by the presence of a common conserved region, the BAG domain at C-terminal, that interacts with ATPase domain of heat-shock protein 70 (HSP70/HSC70) and modulate HSP70 chaperone proteins and form complexes with a different transcription factors^3,9,10^.

Unlike animal counterpart, plant BAG proteins are poorly studied, and the first plant BAG protein was identified and characterized by Doukhanina et al.^11^ in *Arabidopsis*. Later, seven members of *Arabidopsis* BAG protein family have been identified with a BAG domain similar to mammalian BAGs^11^. Four AtBAG proteins (AtBAG1–4) contain a N-terminal ubiquitin like (UBL) domains whereas the remaining three AtBAGs (AtBAG5-7) contain a plant-specific calmodulin-binding domain suggesting a difference between animal and plant PCD pathways and their regulation^3,6,12^. Like animal BAGs, *Arabidopsis* BAGs also act as co-chaperones during environmental stress and plant development^6,11,12^. It is reported that for optimal chaperone activity of BAGs, the ratio of BAG proteins and HSP70 in a cell is crucial. The high BAG proteins and HSP70 ratio negatively affect the refolding activity of HSP70 by modifying its ATP hydrolysis cycle^11,13,14^. The refolding activities of HSP70/HSC70 are critical for cell survival during stressful environments, therefore regulation of or by BAG proteins is vital under such conditions.

Previous studies suggest that BAG proteins are involved in numerous plant pathways including PCD, homeostasis, growth and development, abiotic and biotic stress response^3,6,15^. Several BAG proteins of *Arabidopsis* such as AtBAG4-7 have critical roles in abiotic stress-induced cell death, ROS production and leaf senescence, autophagy during pathogen attack, unfolded protein response for heat and cold tolerance^11, 16–22^. Animal BAGs are localized into either the nucleus or the cytoplasm, but in *Arabidopsis*, BAG proteins are dispersed throughout the cell particularly in mitochondria (AtBAG5), vacuole (AtBAG6), endoplasmic reticulum (AtBAG7), nucleus (AtBAG6 and 7) and cytoplasm (AtBAG1–4)^11,16–24^. The diverse subcellular locations and presence of CaM-binding motifs in several BAG proteins of *Arabidopsis* indicate that plant BAGs may have evolved divergent roles compared with their animal counterparts. As in plants, no homologs of Bcl2 family members and other core animal cell death regulators have been reported, therefore it is still unclear how plant BAG proteins regulate programmed cell death in plants^6^.

Apart from *Arabidopsis,* BAG proteins have also been reported in several other plant species such as rice^25^, maize^26^ and soybean^27^, however, information about BAG proteins in other plants particulary in fruit crops remains elusive. Tomato (*Solanum lycopersicum*) is a fruit model crop with approximately 182.3 million tons annual production (FAOSTAT, 2019), therefore it is quite relevant to identify and characterize tomato BAG proteins. With this aim, we carried out genome-wide identification of tomato BAG gene family followed by gene architecture assessment, conserved domains analysis, exon–intron structure and their chromosomal location. Further, we analyzed the phylogenetic relationship of tomato BAG genes (*SlBAGs*) with other model plant species. In silico analysis of *SlBAGs* promoter regions is also carried out to identify cis-acting regulatory elements. Expression profiling of *SlBAGs* in different plant tissues, during stress conditions and after hormonal treatment was elucidated. In addition to this, we have also carried out subcellular localization and protein–protein interaction study of SlBAGs.

## Results

### Genome-wide identification, gene structure and domain analysis of *SlBAGs*

To identify the BAG domain-containing proteins in tomato, Hidden Markov Model (HMM) profiling of BAG domain (PF02179) and BLASTP search were carried out by using tomato proteome sequence database and Phytozome v12.1. Further, the presence of BAG domain in the identified proteins was validated by using the SMART and Pfam database. A total of 11 proteins were identified in tomato and the nomenclature of these proteins was assigned according to their known *Arabidopsis* homologs followed by their chromosomal location. We observed that chromosome 6 contains 3 genes (*SlBAG2*, *SlBAG6*, *SlBAG11*), followed by chromosomes 3 and 10 (with each 2) and chromosomes 1, 4 and 8 where only one *SlBAG* was located (Table 1).

**Table 1:** Identification, chromosome location and properties of tomato *BAG* domain-containing proteins.

Gene name	Gene ID	Chromosome number	Genomic location	Strand	Exon	Intron	Genomic Size (bp)	CDS (bp)	No. of aa	Protein MW (KDa)	Protein pI
*SlBAG1*	Solyc03g026220.2	3	SL2.50ch03:3630179..3632140	+	4	3	1962	1685	342	38.25	9.52
*SlBAG2*	Solyc06g035720.2	6	SL2.50ch06:24816413..24819781	−	5	4	3369	1492	331	37.24	9.45
*SlBAG3*	Solyc08g080320.2	8	SL2.50ch08:63644732..63647054	−	4	3	2323	1007	277	31.39	9.7
*SlBAG4*	Solyc06g007240.2	6	SL2.50ch06:1298332..1301740	+	4	3	3409	1069	279	31.53	6.28
*SlBAG5*	Solyc04g014740.1	4	SL2.50ch04:5000220..5000519	+	1	0	300	300	100	11.51	4.54
*SlBAG6*	Solyc01g095320.2	1	SL2.50ch01:86628756..86633652	+	5	4	4897	3622	973	108.58	5.44
*SlBAG7*	Solyc03g083970.2	3	SL2.50ch03:53922432..53925268	−	3	2	2837	1661	396	45.28	9.43
*SlBAG8*	Solyc10g085290.1	10	SL2.50ch10:64518728..64523419	+	5	4	4692	1050	350	37.64	5.03
*SlBAG9*	Solyc02g088660.2	2	SL2.50ch02:50673995..50675853	−	2	1	1859	1768	461	51.54	8.64
*SlBAG10*	Solyc10g084170.1	10	SL2.50ch10:63832179..63832691	+	1	0	513	513	171	19.42	10.26
*SlBAG11*	Solyc06g072430.1	6	SL2.50ch06:44683424..44684545	+	1	0	1122	1122	374	42.50	5.66

**Figure 1:**
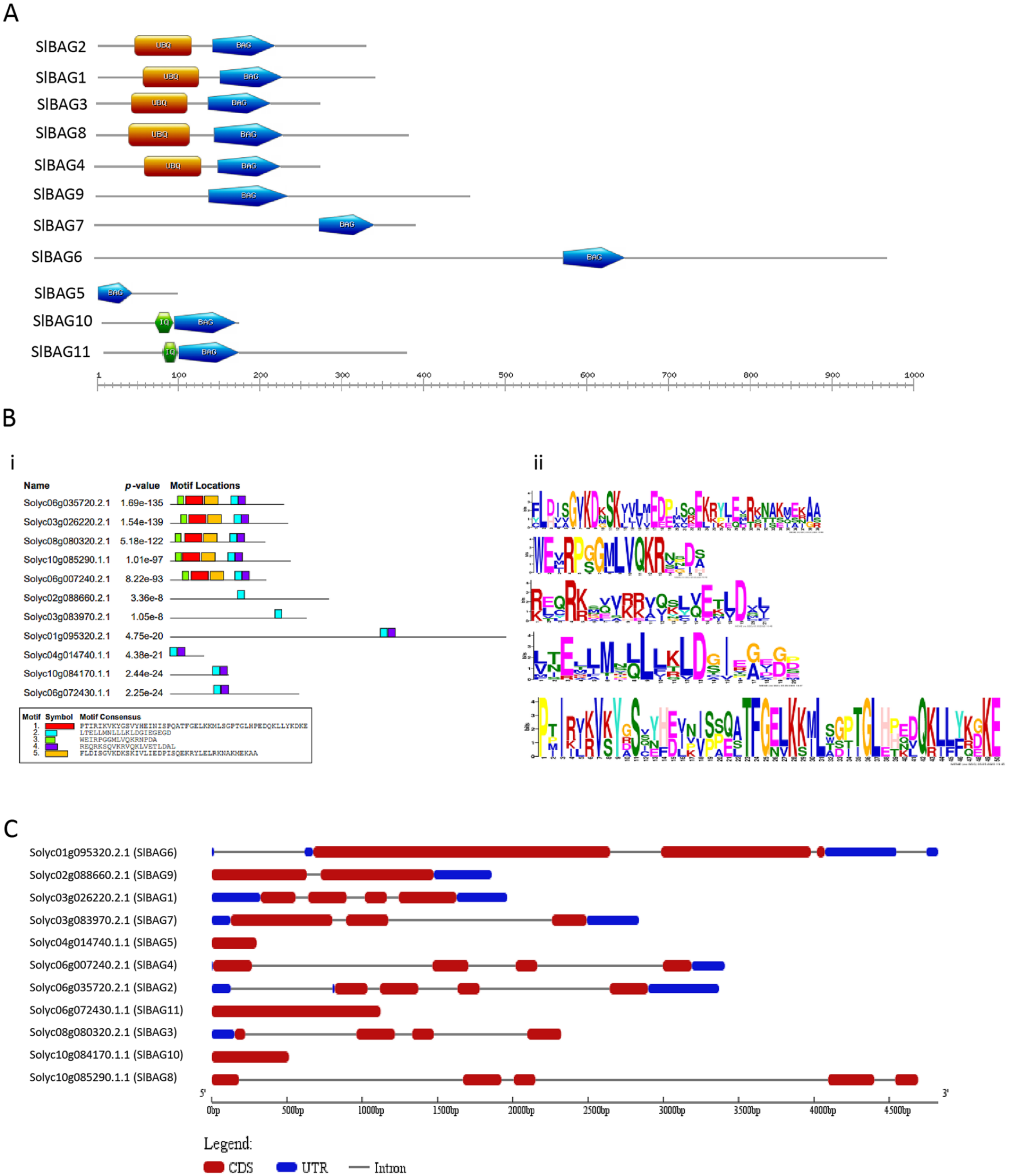
Gene architecture of *SlBAG* family of tomato. (**A**) Domain analysis; The SlBAG protein length can be estimated using the scale at the bottom. (**B**) (i) 5 conserved motifs, identified using MEME Suite 5.4.1 (https://meme-suite.org/meme/); Each motif is displayed in different color (ii) Sequence logos of conserved motifs. The x-axis denotes the width of the motif, and y-axis represents the frequency of each letter (**C**) Exon–Intron structure of *SlBAG* genes. Red and blue rectangles represent the CDS and UTR regions respectively, and black line represents intron. The CDS, UTR and intron length of *SlBAGs* has been displayed proportionally.

To understand the domain architectural feature of SlBAG proteins, domain analysis was carried out using MEME tools. The analysis revealed five conserved motifs in all SlBAG proteins (Fig. 1). We also noticed that a single highly conserved motif in SlBAG proteins belongs to the BAG domain (Fig. 1A,B). The data suggest that SlBAG1-4 and SlBAG8 also contain the UBQ domain and SlBAG10-11 contain IQ domain in addition to the BAG domain (Fig. 1A,B). The exon/intron structure analysis showed that the majority of the BAG genes contain intron except *SlBAG5, SlBAG10* and *SlBAG11* that are intronless genes (Fig. 1C, Table 1). Four genes (*SlBAG5*, *SlBAG8*, *SlBAG10* and *SlBAG11*) do not contain the UTR region whereas *SlBAG3* and *SlBAG9* contain only 5’UTR and 3’UTR respectively (Fig. 1C, Table 1). The remaining *SlBAG* genes contain UTR at both ends. The number of amino acids and molecular weight of BAG proteins varies greatly from 100 amino acids and 11.5 kDa (SlBAG5) to 973 amino acids to 108.58 kda (SlBAG6) (Table 1). The theoretical isoelectric point of six SlBAG proteins was calculated in the basic range and five SlBAG proteins showed acidic pI (Table 1).

### Phylogenetic analysis of SlBAG protein family

**Figure 2:**
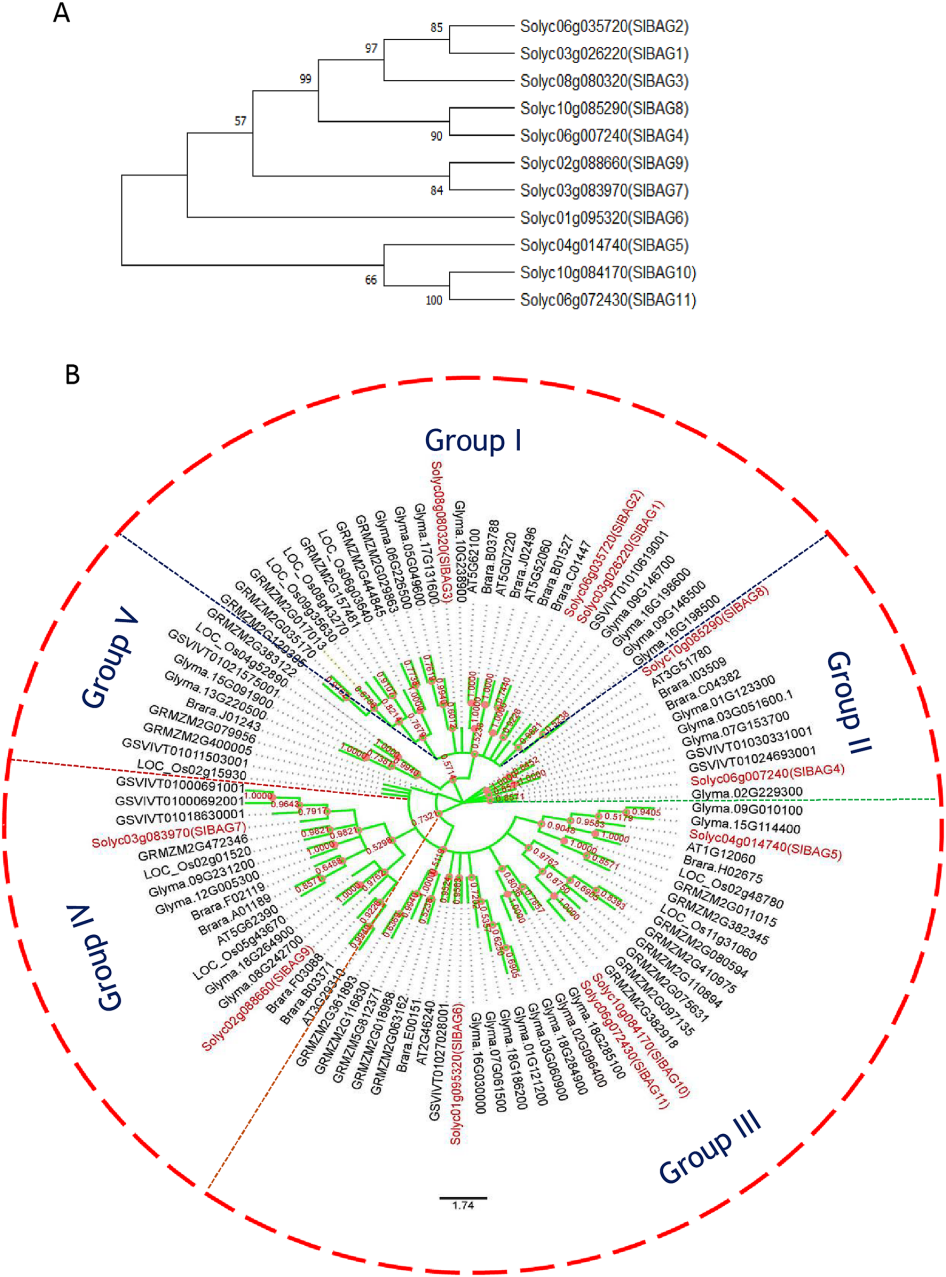
Phylogenetic analysis of SlBAG protein family of tomato and other plant species. (**A**) Evolutionary relationship among SlBAG proteins. (**B**) Phylogenetic tree of SlBAG proteins. The amino acid sequences of BAG family proteins from *Solanum lycopersicon*, *Arabidopsis thaliana*, *Brassica rapa*, *Glycine max*, *Oryza sativa*, *Vitis vinifera* and *Zea mays* were imported into the Molecular Evolutionary Genetics Analysis tool (MEGA X 10.1) (https://www.megasoftware.net/) to make a phylogenetic tree by maximum-likelihood and bootstrap analysis was executed with 1000 replicates/iterations.

The BAG protein family in eukaryotes is highly conserved both in its structure and biological activity^15^. Phylogenetic classification of gene family serves as an important reference to understand functional divergence among the different kingdom of life. To carry out a phylogenetic analysis of tomato BAG*-*domain proteins, we extracted 104 amino acid sequences of BAG proteins from several plant species including both monocots and dicots viz. *Lycopersicon esculentum* (11 BAGs), *Arabidopsis thaliana* (8 BAGs), *Brassica rapa* (13 BAGs), *Glycine max* (28 BAGs), *Oryza sativa* (10 BAGs), *Vitis vinifera* (9 BAGs) and *Zea mays* (25 BAGs) (Table S3). The evolutionary history was inferred by using the maximum likelihood method and JTT matrix-based model^28^. The bootstrap consensus tree inferred from 1000 replicates represented the evolutionary history of the analyzed taxa^29^. Branches corresponding to partitions reproduced in less than 50% bootstrap replicates has been collapsed. Initial trees for the heuristic search were obtained automatically by applying Neighbour-Join and BioNJ algorithms to a matrix of pairwise distances estimated using the JTT model and then selecting the topology with superior log likelihood value. The present analysis resulted in a total of 1645 positions in the final dataset of evolutionary analyses using MEGA X^30^. Our data suggest that BAGs proteins are highly conserved across the plants including both monocots and dicot. Based on phylogenetic analysis, SlBAGs has been categorized into five main groups (group I–V, Fig. 2). Among these groups, a maximum of four proteins (SIBAG5, SIBAG6, SIBAG10, SIBAG11**)** accounted in group III followed by group I (SIBAG1, SIBAG2, SIBAG3), group IV (SIBAG7, SIBAG9) and group II (SIBAG4, SIBAG8). Maximum evolutionary relationship among the BAG proteins were occurred between SIBAG1, SIBAG2 and SIBAG10, SIBAG11 and with other studies taxa and represents ancient eukaryotic BAG-domain proteins with an evolutionary point of view as depicted in Fig. 2.

### In silico promoter analysis of *SlBAGs*

To identify the cis-acting elements involved in the regulation of *SlBAGs* function, we retrieved 2 kb upstream region from the start codon of all *SlBAGs* from the tomato genome sequence database. The in silico analysis of these promoters was carried out by using PlantCARE, PLACE and fuzznuc program of EMBOSS package. The data revealed the occurrence of a large number of different cis motifs in these promoters. The identified cis-acting elements were categorized into three groups naming stress-related elements, hormonal response elements and fruit ripening-related elements based on their functional involvement (Table 2). Interestingly, most of the *SlBAG* promoters except *SlBAG6* and *SlBAG9* contain heat shock elements (HSE) suggesting their possible involvement in heat shock response. *SlBAG1*, *SlBAG2* and *SlBAG9* promoters contain LTR cis-acting elements that are involved in low-temperature responsiveness. MYB transcriptional factor binding sites (MBS) which regulate stress response are also quite common in *SlBAG* promoters and most *SlBAG* promoters except *SlBAG5* and *SlBAG8* contain MBS. We also detected another stress-related motif TC-rich repeat in most *SlBAG* promoters except *SlBAG2* and *SlBAG4*. Further, several motifs related to hormone signaling and regulation were also noticed in the promoter regions of *SlBAG* genes. The most common motif was ABA-responsive elements (ABREs) which were found in eight *SlBAG* promoters followed by ethylene response elements (EREs) in seven *SlBAG* promoters. Other hormonal response elements such as MeJA-responsive elements (CGTCA-motif), salicylic acid-responsive element (TCA), gibberellin-responsive elements (GARE, TATC, P-Box) were also identified. Furthermore, we also reported fruit ripening related elements such as GCC-box and CArG box in the several *SlBAG* promoters. All this data suggests that *SlBAG* genes might be involved in hormonal signaling, plant development and stress response.

**Table 2:** Cis-acting elements located on *SlBAGs* promoters.

Promoter	Stress-related elements	Hormonal-response elements	Ripening-related elements
*SlBAG1*	HSE, MBS, LTR, TC-rich repeats	CGTCA-motif, TCA-element	CArG-Box
*SlBAG2*	HSE, MBS, LTR	ABRE, ERE, CGTCA-motif, TCA-element, TATC-box	–
*SlBAG3*	HSE, MBS, TC rich repeats	ABRE, ERE, P-box, TCA-Element	CArG-Box
*SlBAG4*	HSE, MBS	ERE, GARE-motif, CGTCA-motif, TCA-element	CArG-Box
*SlBAG5*	HSE, TC-rich repeats,	ERE, TCA-element, TATC-box	CArG-Box
*SlBAG6*	MBS, TC-rich repeats	ABRE, P-box, AE-box, CE-3, GARE-motif	CArG-Box
*SlBAG7*	HSE, MBS, TC-rich repeats	ABRE, P-box, TATC-box, TCA-elements	CArG-Box, GCC-box
*SlBAG8*	HSE, TC-rich repeats	ABRE, ERE, GARE-motif	CArG-Box
*SlBAG9*	MBS, LTR, TC rich repeats	ABRE, CGTCA-motif, TCA-Elements	–
*SlBAG10*	HSE, MBS, TC-rich repeats	ABRE, ERE, GARE-motif	–
*SlBAG11*	HSE, MBS, TC-rich repeats	ABRE, ERE, CGTCA-motif, TCA-element	CArG-Box

### Tissue-specific expression analysis

**Figure 3:**
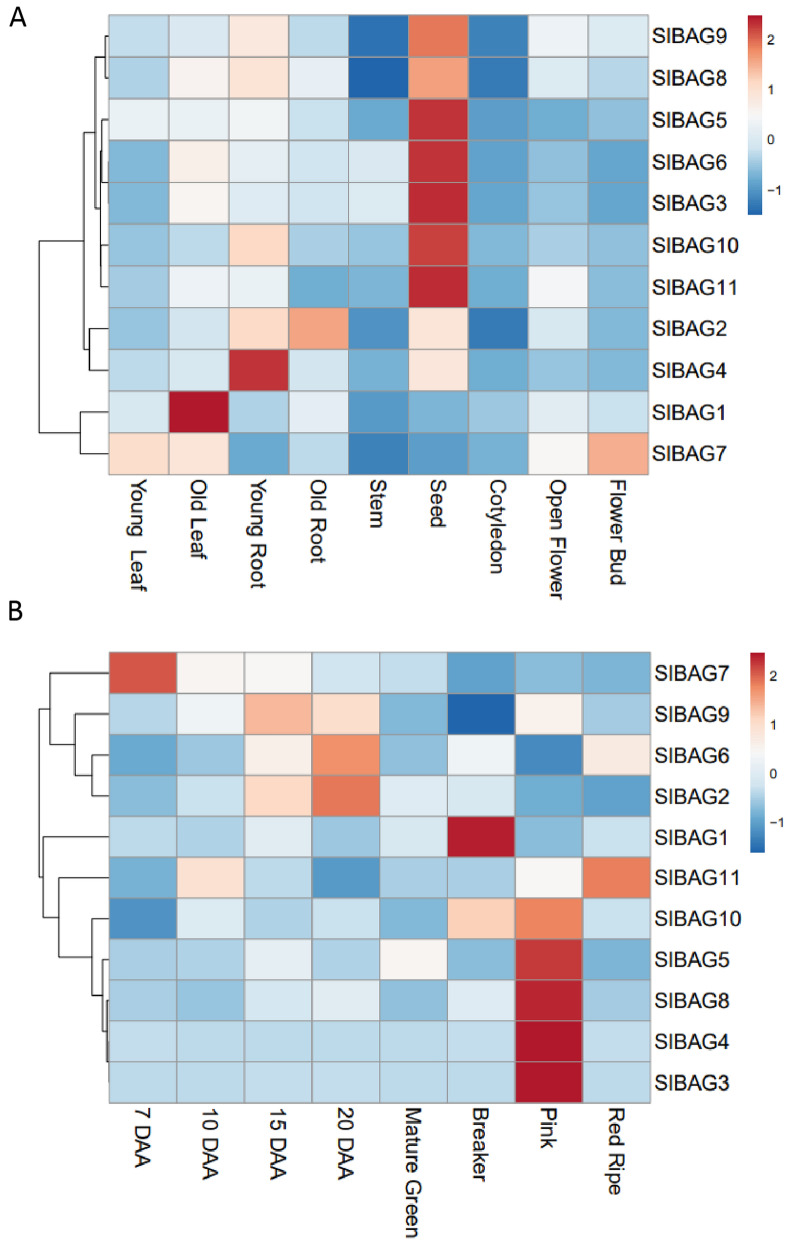
Tissue specific expression analysis of *SlBAGs* performed using qRT-PCR. Transcript accumulation of *SlBAGs* was measured by qRT-PCR using tomato actin as endogenous control. qRT-PCR data was used to make a heat map for displaying gene expression using clustvis tool (https://biit.cs.ut.ee/clustvis/)^48^. (**A**) Heat map showing *SlBAGs* expression during different plant tissues (**B**) Heat map showing *SlBAGs* expression during fruit developmental and ripening stages. After tagging flowers at anthesis, fruits were harvested at 7 to 20 days after anthesis (DAA), mature green, breaker, pink and red ripe stages of fruit development and ripening. Each qRT-PCR reaction was performed with three independent biological replicates, each consisting of three independent technical replicates. The 2^−ΔΔCT^ method for analyzing the qRT-PCR data was employed. The values were converted to log2 fold change in expression before making the heat map.

To gain insights into the biological function of *SlBAG* family genes, spatio-temporal expression analysis of *SlBAG* genes was examined by qRT-PCR using the primers listed in Table S1. The data revealed that seeds showed the highest upregulation of most genes except *SlBAG1* and *SlBAG7* which indicated a slight decrease in their transcript accumulation (Fig. 3A). The expression of the majority of *SlBAG* genes was also downregulated in cotyledonary leaves, young leaves, stem and flower bud. *SlBAG7* expression was elevated in the flower bud as compared to the other genes. While, *SlBAG1, SlBAG2* and *SlBAG4* also showed increased transcript accumulation in the old leaf, old and young root, respectively (Fig. 3A). As we identified several CArG boxes in the promoters of BAG, we carried out expression analysis of *SlBAG* family genes during fruit development and ripening. At seven days after anthesis (DAA), the expression of *SlBAG7* was increased significantly while another *SlBAG* gene, *SlBAG10* showed slightly reduced expression at the same stage (Fig. 3B). During 20 DAA, the transcript accumulation of *SlBAG2* and *SlBAG6* was elevated. Interestingly, we also observed that during fruit ripening stages (breaker, pink and red ripe), several *SlBAG* genes showed significant upregulation in gene expression, for instance, the expression of *SlBAG1* and *SlBAG10* was high during the breaker stage. Similarly, we also noticed increased expression of several other *SlBAG* genes viz*. SlBAG3*, *SlBAG4*, *SlBAG5*, *SlBAG8* and *SlBAG10* during the pink stage and *SlBAG11* during red ripe stage of fruit ripening (Fig. 3B). Few genes such as *SlBAG9* and *SlBAG6* expression goes down during breaker and pink stages, respectively.

### Expression analysis under stress

**Figure 4:**
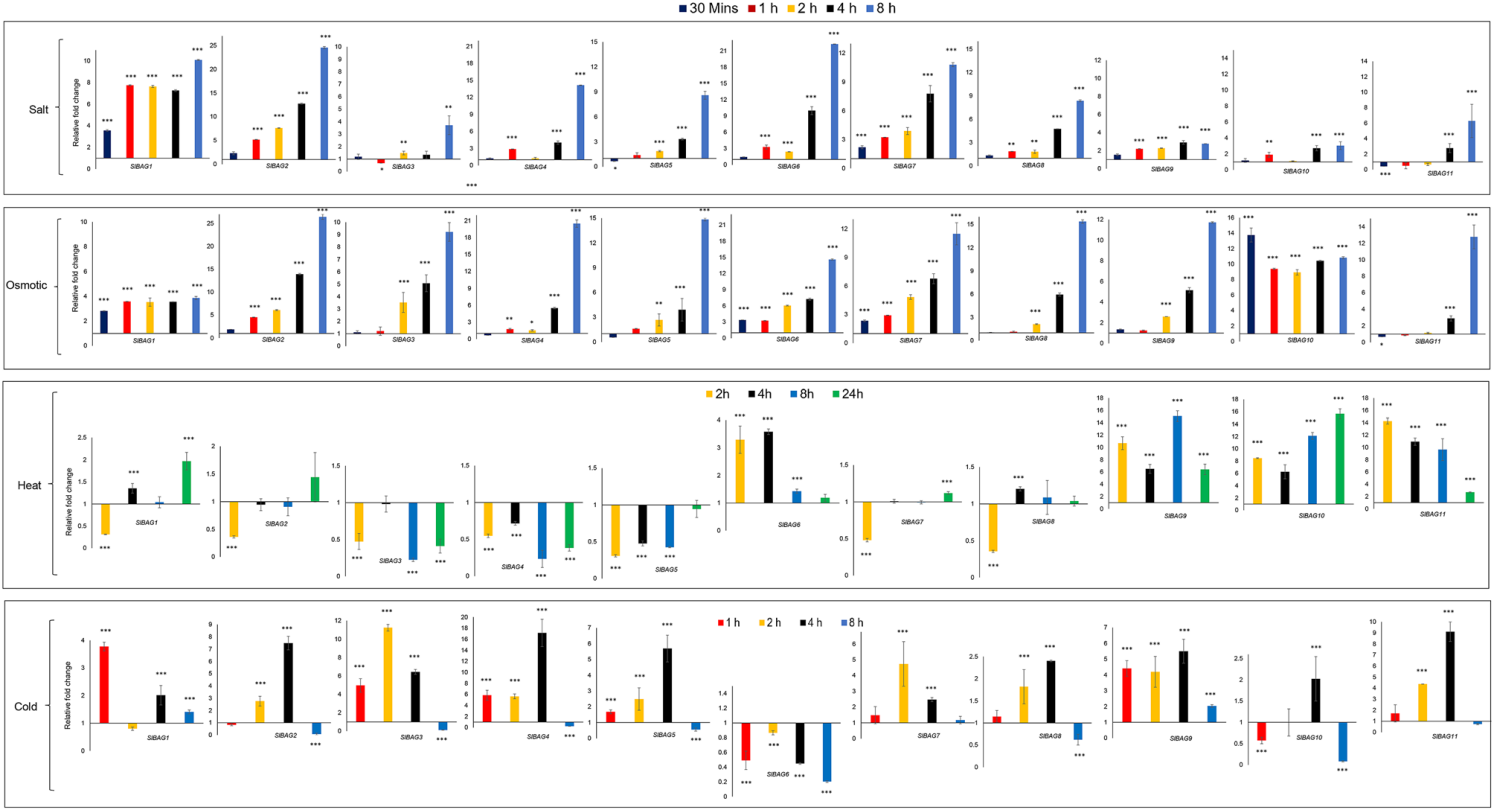
qRT-PCR based expression profiling of *SlBAGs* during abiotic stress conditions. Transcript accumulation in 15-day-old tomato seedlings exposed to 200 mM NaCl for salt stress, 250 mM mannitol for osmotic stress, heat and cold was measured by qRT-PCR using tomato actin as endogenous control by considering reference expression level 1 at 0 h time point using 2^−ΔΔCT^ method. All data are presented as the mean (± SE) of three independent biological determinations and analysed using Student’s t-test (*p < 0.05, **p < 0.01, ***p < 0.001).

Previous studies on *Arabidopsis* BAG proteins have suggested that these proteins are involved in stress response and promoters of *AtBAG* genes contain several stress-related cis-acting elements^15^ therefore expression of *SlBAG* family genes was investigated by qRT-PCR during different abiotic stresses such as salt, osmotic, heat and cold. In response to salt stress, all *SlBAG* genes were upregulated after 30 min of salt treatment to seedlings (Fig. 4). The expression was further increased till 8 h except for *SlBAG11* expression that first showed down-regulation till 2 h and then sixfold upregulation after 8 h salt treatment. During osmotic stress, all *SlBAG* genes except *SlBAG9* and *SlBAG10* exhibited similar gene expression patterns but with different fold as salt stress (Fig. 4). The transcript accumulation of *SlBAG9* was comparatively high (5- and tenfold at 4 and 8 h, respectively). Similarly, *SlBAG10* showed approximately tenfold upregulation after treatment. During heat stress, the expression of *SlBAG1*, *SlBAG2*, *SlBAG3*, *SlBAG4*, *SlBAG5*, *SlBAG7* and *SlBAG8* goes down at 2 h of heat stress as compared to unstressed control (Fig. 4). Further, these genes showed almost the same expression as unstressed plants till 24 h except *SlBAG3* and *SlBAG4* that showed a significant decrease at 8 and 24 h of heat stress. Interestingly, we also observed very high upregulation in the expression of *SlBAG9*, *SlBAG10* and *SlBAG11*. *SlBAG6* also showed > threefold upregulation till 4 h after heat stress followed by almost similar expression as to unstressed plants till 24 h. In case of cold stress or low-temperature treatment, all *SlBAG* genes exhibited increased expression during cold stress except *SlBAG6* that showed downregulation (Fig. 4).

### Expression analysis after hormonal treatment

**Figure 5:**
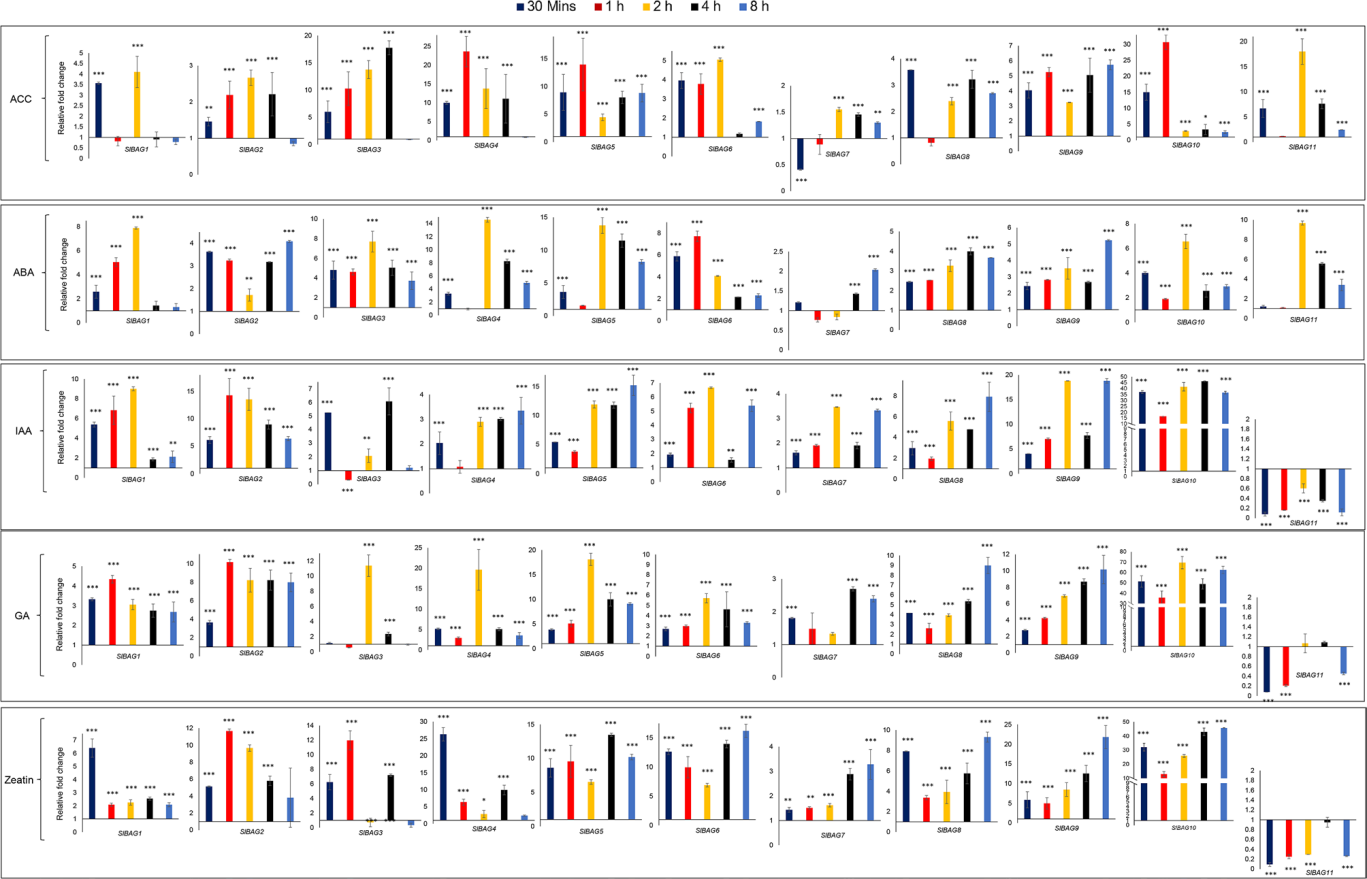
qRT-PCR based expression profiling of *SlBAGs* after hormonal treatment to tomato seedlings. Transcript accumulation in 15-d-old tomato seedlings exposed to 1 mM ACC, 0.1 mM ABA, 0.02 mM IAA, 0.02 mM GA_3_ and 0.02 mM zeatin was measured by qRT-PCR using tomato actin as endogenous control by considering reference expression level 1 at 0 h time point using 2^−ΔΔCT^ method. All data are presented as the mean (± SE) of three independent biological determinations and analysed using Student’s t-test (*p < 0.05, **p < 0.01, ***p < 0.001).

To further characterise the expression pattern of these *SlBAG* genes during plant development and stress response, the tomato seedlings were treated with several hormones such as auxin (IAA), gibberellin (GA-23), zeatin, abscisic acid (ABA), 1-Aminocyclopropane-1-carboxylic acid (ACC, the ethylene precursor) and gene expression analysis was carried out using qRT-PCR. A similar expression pattern of all *SlBAG* genes was observed after auxin, gibberellin, and zeatin treatment (Fig. 5). All *SlBAG* genes except *SlBAG11* which showed downregulation were upregulated after treatment with the above growth hormones. After treating the plants with stress hormones ABA and ethylene precursor ACC, it has been noticed that *SlBAG* exhibited a similar expression pattern and several *SlBAG* genes such as *SlBAG1*, *SlBAG3*, *SlBAG4*, *SlBAG5*, *SlBAG6*, *SlBAG10*, and *SlBAG11* showed very high expression in both treatments (Fig. 5). All this data suggests that *SlBAG* proteins might have critical roles in stress response and plant developmental pathways including fruit development and ripening.

### Subcellular localization of *SlBAG* gene family

**Figure 6:**
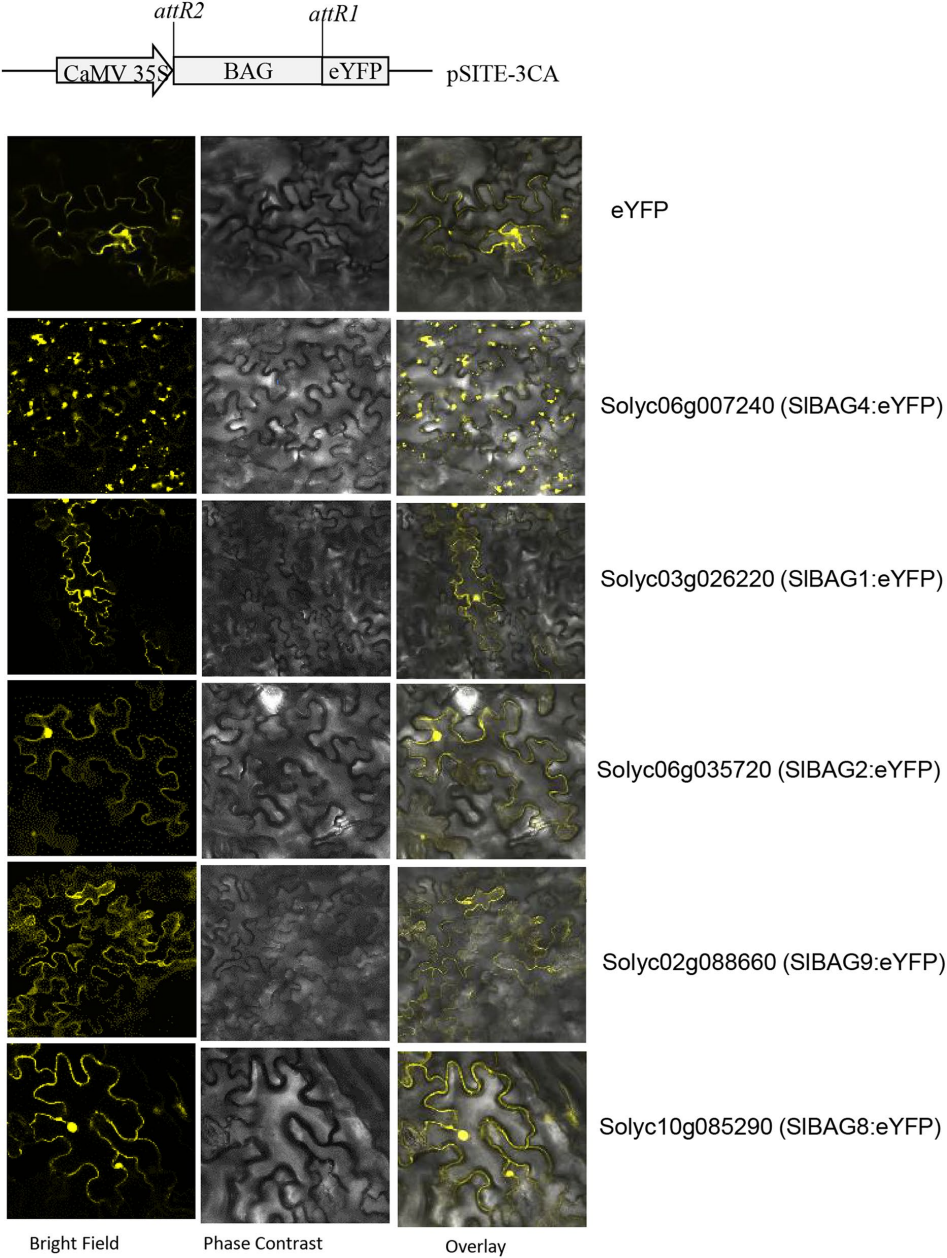
Subcellular localization of eYFP fused SlBAG proteins in *Nicotiana benthamiana.* Confocal microscopy images (Yellow fluorescence, visible light and merged images) were taken from the epidermal cells of leaves infiltrated with *Agrobacterium tumefaciens* GV3101 harbouring vector containing *SlBAG-eYFP* at 60 h post infiltration.

The predicted target signal peptides for SlBAGs proteins were checked using the WoLF PSORT and CELLO that indicated that most proteins were confined in the nucleus, chloroplast and cytoplasm (Table S2). According to CELLO prediction, SlBAGs scored relatively higher in the nucleus except for SlBAG5 which was predicted in cytoplasm and mitochondria (Table S2). WoLF PSORT predicted localization of four SlBAGs (SlBAG2, SlBAG4, SlBAG6, SlBAG9) in the nucleus, four in the chloroplast (SlBAG3, SlBAG5, SlBAG7, SlBAG8), two in the cytosol (SlBAG10, SlBAG11), and one protein (SlBAG1) shared dual locations (nucleus and mitochondria). Several SlBAGs also showed multi-organellar localization in golgi bodies and plastids (dual), cytoplasm and nucleus (dual), cytoplasm, chloroplast, nucleus, mitochondria, peroxisome (Table S2). In total, the differences in the location of SlBAGs were notable between the two prediction programs (Table S2). To confirm the subcellular localization of SlBAGs, several *SlBAG* genes were cloned into pSITE-3CA vector with N-terminal fusion of *eYFP*. These constructs harboring different *eYFP* fused *SlBAG* genes were infiltrated into leaves of *Nicotiana benthamiana* and epidermal cells were observed with a confocal microscope under the YFP channel at 60 h post infiltration. In the case of *SlBAG1, SlBAG2, SlBAG8* and *SlBAG9*, the YFP fluorescence was observed not only in the nucleus but also at the cell periphery which most likely represents a thin layer of cytoplasm that is between the plasma membrane and tonoplast (Fig. 6). Interestingly, *SlBAG4* is expressed as intracellular punctate structures that may represent a golgi apparatus or ER (Fig. 6).

### Interaction of SlBAG proteins with HSPs

**Figure 7:**
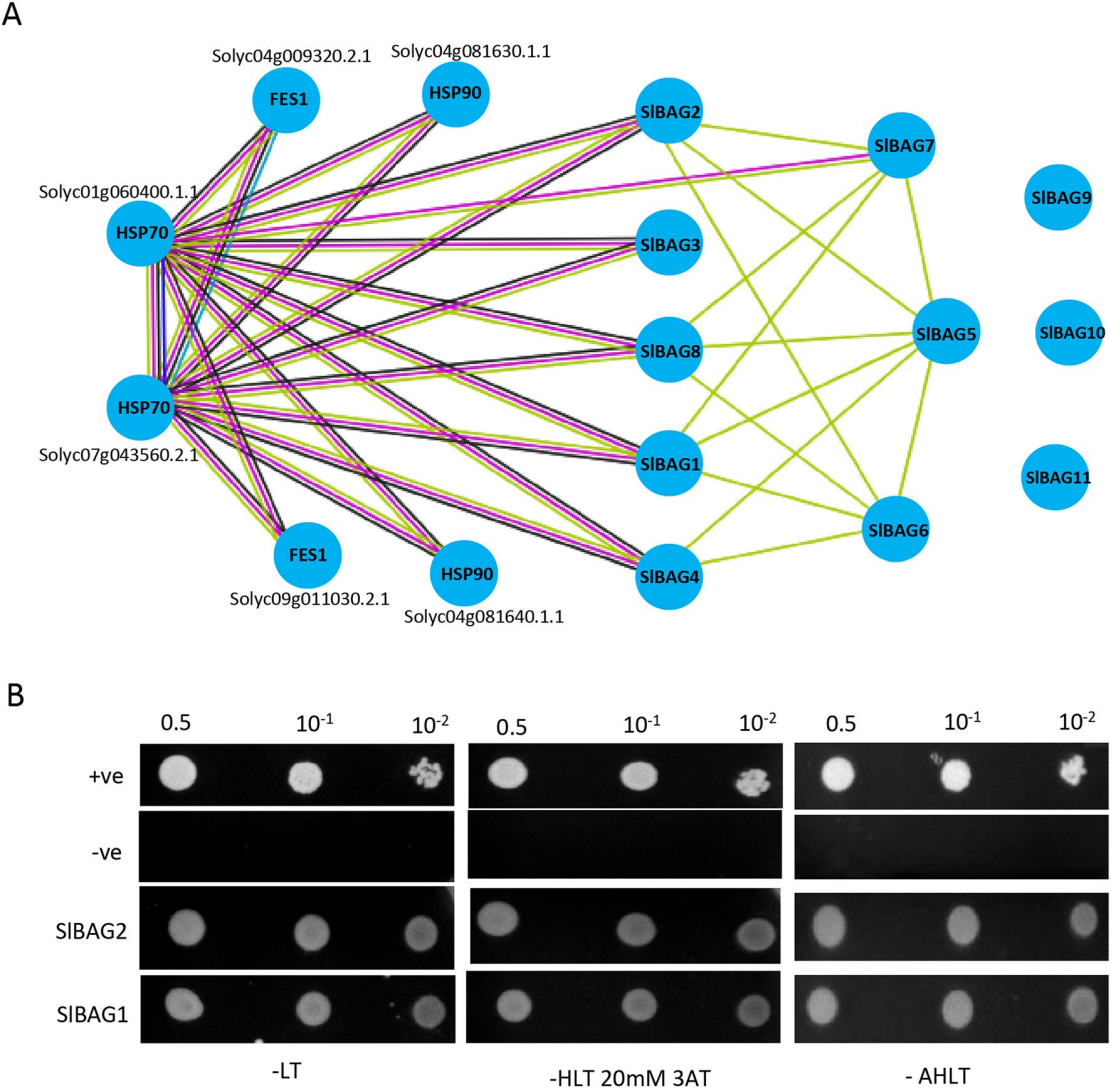
Protein–protein interaction analysis of SlBAG proteins. (**A**) Predictive PPI networks obtained from STRING (https://string-db.org/) with tomato SlBAG proteins. Red line—indicates the presence of fusion evidence; Green line—neighborhood evidence; Blue line—cooccurrence evidence; Purple line—experimental evidence; Yellow line—textmining evidence; Light blue line—database evidence; Black line—coexpression evidence. (**B**) Yeast two hybrid Assay showing interaction of SlBAG1 and SlBAG2 with HSP70 proteins of tomato.

To identify interacting partners of SlBAG proteins, in silico protein–protein interaction (PPI) analysis was carried out using STRING 11.0 database, the most commonly used protein–protein interaction database of 2031 organisms. This database predicts the functional association among proteins based on co-expression and experimental evidence at a global scale. The protein–protein interaction map was developed by using the recommended 0.7 gene interaction/combined score cutoff. The interaction map inferred that SlBAG1, SlBAG2, SlBAG3, SlBAG4, SlBAG8 strongly interact with two HSP70 proteins (Solyc07g043560.2.1 and Solyc01g060400.1.1) (Fig. 7A). The map also suggests that SlBAG7 might also interact with HSP70 protein Solyc01g060400.1.1. Interestingly, few SlBAGs also interacts with themselves to make a complex with HSP70. Moreover, in the PPI map, SlBAG9, SlBAG10 and SlBAG11 did not interact with any proteins (Fig. 7A).

In order to validate the interaction of SlBAG proteins with HSP70, we performed yeast two-hybrid analysis. For this, two candidate proteins SlBAG1 and SlBAG2 were selected and their genes were cloned into prey vector pGADT7-AD separately. Similarly, *HSP70* (Solyc01g060400.1.1) was cloned into a bait vector pGBKT7. Both prey and bait vectors were co-transformed into *Saccharomyces cerevisiae* (strain Y-187) and plated on leucineˉ (Lˉ) and tryptophanˉ (Wˉ) plates. For positive control, pGBKT7-p53 and pGADT7-T were used and for negative control two non-interacting plasmids pGBKT7-Lam and pGADT7-T were used. Colonies obtained on (Lˉ Wˉ) plates showed the double transformation in between the vector pGBKT7 containing TRP1sequence and pGAD containing LEU2 sequence. The colonies that appeared on (Lˉ Wˉ) were streaked on triple dropout media (Lˉ Wˉ Hˉ). On triple dropout plates, all the co-transformants showed colonies including negative transformants which is due to leaky expression of *HIS* genes. To prevent this leaky expression and identification of positive interaction, cells from triple dropout were re-streaked on triple dropout plates containing 5 mM of 3AT. 3AT act as a competitive inhibiter of the His3 protein of yeast cells and suppresses the background growth. In the presence of 3AT, negative control did not grow, whereas positive control grew (Fig. 7B). The cells grown on triple dropout + 3AT showed high stringency in protein–protein interaction. The Fig. 7B shows that SlBAG1 and SlBAG2 interact with HSP70 under different titrations and selection.

## Discussion

BAG domain proteins are highly conserved proteins across animals, plants and microorganisms. However in plants, information on BAG is much less studied and largely *Arabidopsis* BAG family has been identified and characterized^11,15^ apart from several rice, maize and soybean BAG proteins^25–27^. *Arabidopsis* BAG proteins primarily regulate plant developmental pathways and stress response. In this study, we have identified and comprehensively analysed BAG domain-containing proteins of tomato using bioinformatics and wet-lab molecular biology tools. As compared to 7 BAG proteins of *Arabidopsis*, tomato contains 11 BAG proteins suggesting evolutionarily advanced species. Besides tomato, we have also identified BAG proteins in several other plant species (Fig. 2, Table S3) namely *Brassica rapa* (13), *Glycine max* (28), *Oryza sativa* (10), *Vitis vinifera* (9) and *Zea mays* (25). In contrast to previous studies in *Oryza sativa*^25^ and *Zea mays*^26^ where they identified 6 and 13 *BAG* genes respectively, we identified 10 and 25 *BAG* genes in *Oryza sativa* and *Zea mays* respectively most likely due to updated genomic resources. The phylogenetic analysis validates that BAG proteins are highly conserved across the plant kingdom. Based on phylogenetic analysis, tomato BAG-domain proteins have been classified into five groups (Fig. 2). Comparison of intronless genes with a phylogenetic analysis of SlBAGs showed closer genetic relationships to BAG proteins from other dicots species viz. *V. vinifera*, *A. thaliana*, *G. max* than monocots *Zea mays* and *O. sativa*. The results also suggest that plant BAGs of the same categorized group might have similar functions. However, further functional characterization is needed which possibly will provide novel insight for the better understanding of *BAG* genes functions in response to various types of stress, including abiotic stress such as heat, cold, salt, and UV radiation and biotic stress conditions such as hypersensitivity response to pathogens. To understand the functional diversity of SlBAG proteins, domain analysis was carried out using MEME suite which revealed the presence of one highly conserved BAG domain in all the SlBAGs. The presence of N-terminal UBQ domains in several SlBAG proteins such as SlBAG1, SlBAG2, SlBAG3, SlBAG4 and SlBAG8 is similar to other organisms’ BAG proteins. UBQ domains are also found in animal and *Arabidopsis* BAG proteins which are involved in the stress-induced autophagy or ubiquitin–proteasome degradation pathways^11^. Moreover, the presence of IQ calmodulin-binding motif in SlBAG10 and SlBAG11 is quite interesting as this motif is a unique feature of several plant BAG proteins as compared to their animal counterpart^11^. Previously, Doukhanina et al.^11^ have shown that two *Arabidopsis* calmodulin-binding motif-containing BAG genes are specifically induced by Ca^2+^. Several other studies also suggest that plant senescence is regulated by a tripartite signaling complex CaM/AtBAG5/HSC70^21,23^. This suggests that these IQ calmodulin-binding motif BAG proteins may be regulated by calmodulin and possibly by Ca^2+^ and involved in the calcium signaling during PCD and plant stress response.

In order to gain insights into transcriptional regulation, in silico analysis of *SlBAGs* promoters was carried out in search of cis-acting elements. The presence of multiple stress-responsive elements such as HSE, MBS, LTR, TC-rich repeats, ABRE, ERE, GARE, CGTCA-motif indicates possible involvement of *SlBAGs* in plant stress response. This is in accordance with the previous studies on *Arabidopsis BAG* genes that also contain stress-responsive elements including ABRE, ERE, CGTCA motif, MBS, TC-rich repeats^11,15^. Further, the increased GUS activity driven by *AtBAG2* and *AtBAG6* promoters in salt and osmotic stress and post ACC and ABA treatment also suggest that these stress related elements are involved in *BAG* genes regulation during plant stress response^31^. The presence of stress related elements in the *BAG* promoters could be related to stress specific response of *BAG* genes as *BAG* genes from many plants such as *Arabidopsis*, rice, soya bean and wheat have shown plant stress specific response and have been successfully used for improving stress tolerance in *Arabidopsis* and rice^27,31–34^. The potential role of *SlBAGs* in plant stress response is also supported by their differential expression pattern in abiotic stresses and in response to hormonal treatment (Figs. 4 and 5). For instance, the expression of most of *SlBAG* genes including *SlBAG1, SlBAG2, SlBAG4, SlBAG4, SlBAG6* and *SlBAG7* increases under salt and osmotic stress (Fig. 4). The *Arabidopsis* homologues of these genes viz* AtBAG2, AtBAG3, AtBAG4, AtBAG6* also showed increased expression in salt stress conditions^11,15,31^. Moreover, the enhanced expression of *OsBAG4* in rice roots and *TaBAG2* in wheat in response to saline was also reported indicating the involvement of *SlBAGs* in salt stress response^32, 34^. In our data, the pattern of most *SlBAGs* expression during salt and osmotic stress is similar (Fig. 4) and is in accordance with prior *AtBAG* studies^11,15,31^, however the possibility of osmotic shock using 200 mM NaCl cannot be neglected therefore long-term salt treatment assays with gradual NaCl application instead of single step application might provide a clearer picture of *SlBAGs* expression under salt stress^35^.

In the present study, *SlBAG6, SlBAG9, SlBAG10* and *SlBAG11* were induced during heat stress (Fig. 4). Several previous studies suggest the significant upregulation of *AtBAG6* and *AtBAG7* and down regulation of *AtBAG1* and *AtBAG4* in heat stress^11,15,21,23,31^. We also reported downregulation of *SlBAG1* and *SlBAG4* at early time points of heat stress, however we did not report any significant diffence in the *SlBAG7* expression during heat stress. During cold stress, most *SlBAGs* except *SlBAG6* get induced suggesting *SlBAGs* role in cold stress response. In *Arabidopsis*, *AtBAG4* has been induced by cold temperature and transgenic tobacco plants overexpressing *AtBAG4* showed cold tolerance^11^. Moreover, most of the *SlBAGs* genes were upregulated by stress hormones ABA and ethylene precursor ACC (Fig. 5) advocating *SlBAGs* involvement in the abiotic stress response of plants.

Tissue-specific expression profiling of gene families can provide vital clues about their functional differentiation in plant tissue and organs. To explore the expression pattern of the tomato BAG gene family, we carried out the qRT-PCR analysis of *SlBAG* genes during plant developmental stages. The data suggest that expression patterns of *SlBAG* genes varied during different developmental stages of tomato. Most of the *SlBAG* genes exhibited differential expression in seeds which is quite interesting as *SlBAG* genes may regulate seed-specific traits in tomatoes. This is further supported by another study by Doukhanina et al.^11^ in which they identified a BAG EST (AI960691 or Glyma.01G123300.1) in the immature seed coats of soybean^3^. This soybean *BAG* is evolutionarily closer to *SlBAG8* which also showed seed-specific expression (Figs. 2 and 3A). In our prior study, we have shown that *SlBAG1* expression increases during fruit ripening and this gene is involved in geraniol mediated plant senescence^36^, therefore we carried out expression analysis of all *SlBAG* genes during fruit development and ripening. Interestingly, *SlBAG1, SlBAG3, SlBAG4, SlBAG5, SlBAG8, SlBAG10* were upregulated, while *SlBAG6* and *SlBAG9* were downregulated during ripening (Fig. 3B) suggesting their involvement in the fruit ripening, a protracted form of senescence. This ripening-related role of *SlBAG* genes is also supported by the presence of CArG boxes in the promoter regions of *SlBAG* genes (Table 2). RIN, the master regulator of fruit ripening transcriptionally regulates the ripening-related genes by binding to CArG boxes of promoters of these genes^37–39^. In our study, several *SlBAG* genes (*SlBAG1*, *SlBAG3*, *SlBAG4*, *SlBAG5*, *SlBAG6*, *SlBAG10*, *SlBAG11*) are also induced by ACC, the precursor of ripening hormone ethylene and ABA which suggest that *SlBAG* genes are potentially involved in the fruit ripening regulation and stress response. Furthermore, several *SlBAG* genes such as *SlBAG2*, *SlBAG6*, *SlBAG7*, and *SlBAG10* also showed differential expression during fruit development which suggests that they might have a role in fruit development as well. Plant hormones such as auxin, GA and cytokinin regulates fruit development in climacteric and non-climacteric fruit crops^40,41^. The present study has shown that all *SlBAG* genes except *SlBAG11* are upregulated by IAA, GA, and zeatin suggesting that *SlBAG*s may regulate fruit development in tomato.

The study of subcellular localization of proteins is helpful in investigating the biological functions of proteins. In this study, the subcellular localization analysis revealed the varied subcellular localization of SlBAG proteins. For instance, SlBAG2, SlBAG8 and SlBAG9 proteins are mainly localized in the nucleus and cytoplasm, while SlBAG4 is expressed in the mitochondria, golgi, or ER. In previous studies, *Arabidopsis* BAG proteins have also shown the diverse subcellular distribution such as AtBAG1-3 are localized in cytoplasm and AtBAG4 in cytoplasm together with nucleus^15^. Like the previous studies on Arabidopsis, AtBAG5 and AtBAG6 which are reported to localize in mitochondria and vacuole to regulate the organelle-regulated cell death^11,42^. We also observed the similar punctate expression of one of the BAG proteins, SlBAG4 suggesting a similar kind of mechanism of SlBAG4 function might exists in tomato to regulate the intracellular cell death. Recently, it has been reported that under heat and cold stress, ER-localized AtBAG7 translocate to the nucleus in order to regulate the ER-nucleus stress-signaling pathway or unfolded protein response (UPR) pathway^20^. As several *SlBAG* genes also showed differential expression during the heat and cold stress it is possible that during stress SlBAGs might regulate the UPR pathway by interacting with HSP70. In the present investigation, it has been observed that localization prediction of BAG proteins by WoLF PSORT and CELLO tools did not support the actual subcellular localization for some BAG proteins. For instance, in WoLF PSORT and CELLO programs, BAG4 was predicted to primarily localized in the nucleus but actually, it is not localized in the nucleus. Similarly, WoLF PSORT predicted the SlBAG8 localization in chloroplast whereas we observed the SlBAG8 expression in the nucleus and cytoplasm. One of the reasons for this discrepancy could be that some unidentified nuclear localization signals which are not deposited in these databases might exist in the BAG proteins.

As the BAG domains interact with HSP70/HSC70 proteins to modulate the chaperone activity therefore we carried out protein–protein interaction network analysis of SlBAGs which may provide the functional information of these proteins for uncovering the molecular mechanisms of their stress response. The interaction map shows that several SlBAG proteins such as SlBAG1, SlBAG2, SlBAG3, SlBAG4, SlBAG7, SlBAG8 clearly interacts with two of the HSP70 proteins (Solyc07g043560.2.1 and Solyc01g060400.1.1). STRING PPI network also showed that other BAG proteins such as SlBAG5 and SlBAG6 do not interact with HSP70 directly but they interact with other BAGs that interact with HSP70. This suggests that SlBAG5 and SlBAG6 recruit the HSP70 via interacting with SlBAG1, SlBAG2, SlBAG4 and SlBAG8 to form complexes with signaling molecules and molecular chaperones. In the PPI network, we did not observe the interaction of SlBAG9, SlBAG10 and SlBAG11 partially because the STRING database is not always up-to-date, and therefore, they do not show up the interaction. To further validate the interaction of SlBAG2 with HSP70, the yeast two-hybrid analysis was carried out which revealed that SlBAG2 clearly interact with HSP70 protein. All these PPI data suggest that BAG domains allow the BAG-family proteins to function as adapter molecules by recruiting HSP70/HSC70 to regulate the activity of target proteins in order to regulate the stress response and plant development pathways. Therefore, the ectopic expression of *SlBAG* genes together with HSP70 genes in plants could be a novel strategy to confer the stress tolerance for sustainable agriculture. A similar approach has been employed by Hoang et al., (2015) to improve the salt stress tolerance in rice by expressing three anti-apoptotic genes *AtBAG4*, *HSP70* and *p35* from different oroganisms.

## Conclusions

In this study, tomato *BAG* family genes and their encoding proteins were identified by genome-wide screening of tomato genome and proteome. A total of 11 *SlBAG* genes/proteins were identified in tomatoes and classified into five groups based on their phylogenetic relationship. Gene structure, domain analysis, physico-chemical properties and in silico promoter analysis have also been discussed. Present results highlighted that these proteins interact with HSP70 proteins and are primarily located in the cytoplasm and nucleus where they can regulate the activity of target proteins via forming a complex with HSP70 during the plant stress response and plant development. The present data also shows that all *SlBAG* genes showed differential expression patterns during plant development including fruit development and ripening suggesting their potential role in the above processes. Furthermore, these *SlBAG* genes are stress-responsive and exhibited modulation in their expression pattern under various abiotic stresses including heat and cold stress. Taken together all our data, we identified several novel fruits ripening specific and stress stress-responsive *SlBAG* genes. The functional characterization of these *SlBAG* genes would be interesting to understand their interaction with signaling pathways during plant development, senescence and production of crops with enhanced stress tolerance and improved fruit quality traits.

## Materials and methods

All the methods were carried out in accordance with relevant guidelines and regulations.

### Identification and sequence analysis of SlBAG proteins/genes

The identification of tomato BAG proteins was carried out by using BLASTP tool of tomato proteome sequence database (solgenomics.net) and Phytozome v12.1 (https://phytozome.jgi.doe.gov). *Arabidopsis thaliana* BAG proteins sequences (retrieved from Phytozome database) were used as queries to identify the SlBAG homologues in tomato. Further, HMM profile of BAG domain (PF02179) was also used for the identification of SlBAG using HMMER3.0 with an E-value setting of 1e-5. The HMM model of BAG was used as a query to search for all possible BAG protein sequences in the tomato proteome database using BLASTP (E < 0.001). Further, the presence of BAG domain in the SlBAG proteins was confirmed using SMART (http://smart.embl-heidelberg.de/) and Pfam database (http://pfam.xfam.org/). The analysis of identified *SlBAGs* genes and their chromosomal locations were performed by using the BLASTN search tool at the tomato genome sequence database.

### Gene architecture and domain analysis

The conserved motifs in SlBAG proteins were analyzed using MEME (http://meme-suite.org/tools/meme) with default parameters except for the maximum number of motifs was set to 5. The visualization of gene structure was carried out using GSDS software^43^. The physical and chemical properties such as molecular weight, amino acid composition, and theoretical isoelectric point (pI) were predicted by online tool ProtParam (https://web.expasy.org/protparam/).

### Multiple sequence alignment and phylogenetic analysis

The amino acid sequences of BAG family proteins from *Lycopersicon esculentum*, *Arabidopsis thaliana*, *Brassica rapa*, *Glycine max*, *Oryza sativa*, *Vitis vinifera* and *Zea mays* were retrieved from phytozome database and examined together. A complete set of protein sequences were imported into the Molecular Evolutionary Genetics Analysis tool (MEGA X 10.1) and multiple sequence alignment (MSA) was performed through *MUSCLE* (*MUltiple Sequence* Comparison by Log-Expectation) tool with default parameters^44^. The alignment file was imported to construct the phylogenetic tree by maximum-likelihood and bootstrap analysis was executed with 1000 replicates/iterations. The constructed phylogenetic tree was modified using Inkscape software (https://inkscape.org/).

### In silico promoter analysis

The 2 kb upstream sequences from the start codon of all *SlBAG* genes were retrieved from the tomato genome sequence database (www.solgenomics.net). These promoter sequences were analysed by using plantCARE database (http://bioinformatics.psb.ugent.be/webtools/plantcare/html/) for the identification of cis-acting elements. The CArG boxes in the promoters were searched using the FUZZNUC program included in the EMBOSS package (https://www.bioinformatics.nl/cgi-bin/emboss/help/fuzznuc)^45^.

### Plant material and growth conditions

*Solanum lycopersicum* (cv. Pusa ruby) seeds, purchased from National Seeds Corporation Ltd., New Delhi, India were germinated in pre-sterilized soil. The 15-day-old seedlings were transplanted into pots containing agropeat and vermiculite (2:1 v/v) and grown in a plant growth chamber at 25 °C/22 °C day/night temperature, 65% relative humidity and 16/8 h light/dark regimen. For tissue-specific expression analysis, different plant tissues such as leaf, root, flower, flower bud, stem and roots were harvested and stores at − 80 °C until further use. Moreover, tomato fruits were also harvested at different developmental stages [7DAA, days after anthesis), 10DAA, 15DAA and 20DAA] and four ripening stages mature green (MG), breaker (Br), pink (P), and red ripe (RR)] after tagging of flowers at anthesis.

### Hormonal and stress treatment

Tomato seedlings were grown on MS media in magenta boxes for 15 days. These 15-d-old seedlings were transferred to fresh magenta boxes containing liquid MS media with either of 1 mM ACC, 0.1 mM ABA, 0.02 mM IAA, 0.02 mM GA_3_ and 0.02 mM zeatin and incubated for 8 h. The seedlings were harvested in triplicates for each hormonal treatment at 0 h, 2 h, 4 h, and 8 h and frozen immediately in liquid nitrogen. For salt and osmotic stress, the seedlings were treated in a same way as described above with 200 mM NaCl and 250 mM mannitol and tissues were harvested at different time points till 8 h. For heat stress, the plants were kept at 42 °C and tissue was harvested at 0 h, 2 h, 4 h, 8 h and 24 h. The cold stress was given to plants by transferring them to 4 °C followed by tissue harvesting at 0 h, 1 h, 2 h, 4 h and 8 h. All the harvested samples were flash-frozen and stored at − 80 °C until further use.

### RNA isolation and qRT-PCR analysis

The total RNA from different plant tissues was isolated as described previously^46^ followed by purification with RNeasy Mini Kit (Qiagen). Further, to remove the genomic DNA contamination, the RNA samples were treated with DNase I (Invitrogen). The purified RNA samples were quantified by using Nanodrop1000 (Thermo Fisher Scientific, Wilmington, DE, USA) and 5 μg RNA was reverse transcribed to cDNA using superscript II RT (Invitrogen) at 42 °C for 50 min in 20 μl reaction volume following the manufacturer’s instructions. qRT-PCR was performed using ABI Prism 7500 Detection System (Applied Biosystems, Foster City, CA, USA) with SYBR Green dye as described by Kumar et al.^39^. The data are presented as fold change in gene expression, normalized to the endogenous reference gene (actin) and relative to control by using the 2^−ΔΔ CT^ method^47^. The analysis is conducted in triplicate from cDNA derived from at least three independent experiments. Obtained data was dislayed in a heatmap generated by using Clustvis tool^48^. The oligonucleotide primers used for qRT-PCR are listed in Table S1.

### Subcellular localization

In silico subcellular localization of SlBAG family proteins was predicted by WoLF PSORT^49^ and CELLO^50^. For *in planta* subcellular localization, ORF of *SlBAG* genes was cloned into pSITE-3CA with N-terminal eYFP under CaMV35S promoter. *Agrobacterium* culture harboring the pSITE-3CA-*SIBAG* construct was infiltrated into the leaves of *Nicotiana benthamiana* followed by incubation of plants in dark at 25 °C for 2 days. The sections of infiltrated leaves were observed with a Confocal Laser Scanning Microscope Confocal microscope (Leica TCS-SP2) under the YFP channel.

### Protein–protein interaction and yeast two hybrid assay

The Protein–Protein Interaction analysis of SlBAG proteins was carried out by using STRING, a database of known and prediction-based protein–protein interactions^51^. The yeast two hybrid (Y2H) assay was performed by using the Matchmaker two-hybrid system (Clontech Laboratories, Inc.). The sequence of HSP70 (Solyc01g060400.1.1) was obtained from the tomato genome sequence database and fused with GAL4 DNA binding domain of bait vector pGBKT7 by using In-Fusion HD Cloning kit (Takara Bio Inc., Shiga, Japan). Similarly, two SlBAG1 and SlBAG2 were fused with the activation domain of the pGADT7 vector. For positive control, two strongly interacting proteins p53 cloned in pGBKT7 (pGBKT7-p53) and simian virus large T antigen cloned in pGADT7 (pGADT7-T) was used. Two non-interacting plasmids, pGBKT7-Lam and pGADT7-T were also used as a negative control. For Y2H, *S. cerevisiae* (strain Y-187) cells were co-transformed with pGAD-SlBAG1 or pGAD-SlBAG2 along with pGBKT7-HSP70. The transformants were then plated on the medium lacking histidine, leucine and tryptophan (− HLT).

## Supplementary Information

Supplementary Information.
