# Author Correction: Unraveling the role of tomato Bcl-2-associated athanogene (BAG) proteins during abiotic stress response and fruit ripening

**DOI:** 10.1038/s41598-022-07666-7

**Published:** 2022-02-25

**Authors:** Mohammad Irfan, Pankaj Kumar, Irshad Ahmad, Asis Datta

**Affiliations:** 1 National Institute of Plant Genome Research, Aruna Asaf Ali Marg, New Delhi, 110067 India; 2 Plant Biology Section, School of Integrative Plant Science, Cornell University, Ithaca, NY USA; 3 Department of Biotechnology, Dr. Y.S. Parmar University of Horticulture and Forestry, Solan, Himachal Pradesh India

Correction to: *Scientific Reports* 10.1038/s41598-021-01185-7, published online 05 November 2021

The Acknowledgments section in the original version of this Article was incomplete.

“This study was funded by core research grant from National Institute of Plant Genome Research. MI acknowledges CSIR, India for Senior Research Associateship (CSIR Pool Number: 8862-A).”

now reads:

“This study was funded both by core research grant from National Institute of Plant Genome Research and Department of Biotechnology, Ministry of Science and Technology, Government of India. MI acknowledges CSIR, India for Senior Research Associateship (CSIR Pool Number: 8862-A).”

The original Article has been corrected.

