# Supplementary material for: Unraveling the role of tomato Bcl-2-associated athanogene (BAG) proteins during abiotic stress response and fruit ripening
Source: Sci Rep. 2021 Nov 5;11:21734. doi: 10.1038/s41598-021-01185-7 (PMC8571320; doi:10.1038/s41598-021-01185-7)
Supplement: Supplementary file 1 — Supplementary Information. [file 41598_2021_1185_MOESM1_ESM.docx]

**Supplementary material**

**Unraveling the role of tomato Bcl-2-associated Athanogene (BAG) proteins during abiotic stress response and fruit ripening**

Mohammad Irfan^1,2,*^, Pankaj Kumar^1,3,#^, Irshad Ahmad^1,#^, Asis Datta^1,*^

^1^National Institute of Plant Genome Research, Aruna Asaf Ali Marg, New Delhi-110067, India

^2^Plant Biology Section, School of Integrative Plant Sciences, Cornell University, Ithaca, New York, USA

^3^CSIR-Institute of Himalayan Bioresource Technology, Palampur, India

^#^ Contributed equally

*correspondence: [asis_datta@rediffmail.com](mailto:asis_datta@rediffmail.com); mi239@cornell.edu

Supplementary Table S1: List of primers used in this study

Supplementary Table S2: *In silico* subcellular localization prediction of tomato SlBAG proteins

Supplementary Table S3. The list of *BAG* family genes *Arabidopsis thaliana, Brassica rapa, Oryza sativa, Glycine max, Vitis vinifera* and *Zea mays* and their chromosomal location, and the characteristics of encoded proteins.

**Table S1: List of primers used in this study for qRT-PCR Analysis**

| **SN** | **Primer Name** | **Sequence (5’-3’)** | **Amplified gene** |
| --- | --- | --- | --- |
| 1 | RT-Sl80320F | CAGAGACTTTGTTATTGAGTTTGATTGA | *SlBAG3* |
| 2 | RT-Sl80320R | TCACCTGCATTCTTCTCTGTAATTTAA |  |
| 3 | RT-Sl88660F | AAGCCAACAAAGTAACAGACCAACA | *SlBAG9* |
| 4 | RT-Sl88660R | CAGCGATCCGACGGAGAA |  |
| 5 | RT-Sl95320F | TGAAACGCCAGGCAAGGA | *SlBAG6* |
| 6 | RT-Sl95320R | TCTTTTGGCTTATTTGCTGCTTT |  |
| 7 | RT-Sl14740F | GATTTCTAATGGATTGGGATGATGTT | *SlBAG5* |
| 8 | RT-Sl14740R | CCGCCACCTCTCTCTTTGC |  |
| 9 | RT-Sl35720F | GAGCTGAGGCCCAGTGGAA | *SlBAG2* |
| 10 | RT-Sl35720R | TAGTAGGAGGAGGACGACGATTTT |  |
| 11 | RT-Sl85290F | GAGGAAGAAATGGTTGAGGAAAAA | *SlBAG8* |
| 12 | RT-Sl85290R | TCGGCTCCTCTTGATATTTCAGT |  |
| 13 | RT-Sl84170F | ACAATCGGTCCACGAGAAATCT | *SlBAG10* |
| 14 | RT-Sl84170R | GACCGGAATCTGAACGACCTT |  |
| 15 | RT-Sl07240F | GGAAGATCCAGCCAGCAAAG | *SlBAG4* |
| 16 | RT-Sl07240R | CGGGCGATGGCTTGAG |  |
| 17 | RT-Sl26220F | TCAGCAGCATCAGCGTAGGT | *SlBAG1* |
| 18 | RT-Sl26220R | CCTGCTGCCGTTCTGGTTT |  |
| 19 | RT-Sl83970F | CACCCCCAAAACCCTACTTCTA | *SlBAG7* |
| 20 | RT-Sl83970R | CGAGAGTGCAGTCAAGCTCATC |  |
| 21 | RT-Sl72430F | AATCCGATGAACTCTCTGAACCA | *SlBAG11* |
| 22 | RT-Sl72430R | TTTTCCATCTTCATCCTGTTTTCC |  |
| 23 | RT-TAF | TTATCACCATTGGTGCTGAG | Actin (endogenous control) |
| 24 | RT-TAR | CGATGTTTCCATACAGATCCTT |  |

**Table S2: *In silico* subcellular localization prediction of tomato SlBAG proteins**

| **Protein** | **Subcellular localization prediction**  **CELLO^a^ WoLF PSORT^b^** | |
| --- | --- | --- |
| SlBAG1 | nucl (4.3) | nucl (5.5), mito (5), cyto_nucl (4), cyto (1.5), chlo (1), plas (1) |
| SlBAG2 | nucl (3.5) | nucl (7.5), cyto_nucl (5.5), cyto (2.5), chlo (1), plas (1), extr (1), cysk (1) |
| SlBAG3 | nucl (2.6), mito (1.2) | chlo (7), nucl (2), vacu (2) pero (2), golg_plas (1) |
| SlBAG4 | nucl (2.8), cyto (1.3) | nucl (6), cyto (5), chlo (1), mito (1), golg_plas (1) |
| SlBAG5 | nucl (1.7), mito (1.2) | chlo (7), cyto (3), nucl (2), extr (2) |
| SlBAG6 | nucl (4.3) | nucl (11), pero (3) |
| SlBAG7 | nucl (2.9), cyto (1.2) | chlo (4), golg (4), vacu (3), mito (2), nucl (1) |
| SlBAG8 | nucl (1.7) | chlo (5), cyto (3), nucl (2), mito (2), extr (1), vacu (1) |
| SlBAG9 | nucl (4.4) | nucl (9), chlo (2), cyto (1), vacu (1), pero (1) |
| SlBAG10 | nucl (1.9), mito (1.1) | cyto (5), nucl (3), plas (2.5), chlo (2), golg_plas (2), mito (1) |
| SlBAG11 | nucl (3.0), mito (1.0) | cyto (5), nucl (4), chlo (3), mito (1), golg_plas (1) |

The numbers in parenthesis indicate prior possible protein localization sites of the *SlBAGs*. Abbreviations of protein localization sites in the dataset are as follows: nucl- nucleus; chlo- chloroplast; cyto- cytosol; cysk- cytoskeleton; plas- plastids; vacu-vacoule; golg- golgi bodies; extr- extracellular; cyto_nucl-cytosol and nucleus; mito-mitochondria; pero- peroxisomes; and golg_plas- golgi bodies and plastids

**Table S3.** The list of *BAG* (Bcl-2 athanogene) family proteins from *Arabidopsis thaliana*, *Brassica rapa*, *Oryza sativa*, *Glycine max*, *Vitis vinifera* and *Zea mays* and their chromosomal location, and the characteristics of encoded proteins.

| **Locus name** | **Chr** | **Position (start)** | **Position (end)** | **Length (aa)** | **Size (kDa)** | **pI** |
| --- | --- | --- | --- | --- | --- | --- |
| ***Arabidopsis thaliana*** | | | | | | |
| *AT5G52060* | Chr5 | 21152214 | 21154244 | 326 | 36.4 | 8.93 |
| *AT5G62100* | Chr5 | 2494039 | 24941955 | 232 | 25.5 | 8.92 |
| *AT5G07220* | Chr5 | 2265256 | 2266967 | 303 | 34.2 | 9.24 |
| *AT3G51780* | Chr3 | 19206768 | 19208310 | 269 | 29.2 | 5.2 |
| *AT1G12060* | Chr3 | 4076334 | 4076981 | 215 | 24.5 | 9.05 |
| *AT2G46240* | Chr2 | 19206768 | 19208310 | 1043 | 116.8 | 5.2 |
| *AT5G62390* | Chr5 | 25051978 | 25054289 | 446 | 51.6 | 9.28 |
| *AT3G29310* | Chr3 | 11249652 | 11251820 | 551 | 61.4 | 6.92 |
| ***Brassica rapa*** | | | | | | |
| *Brara.E00151* | ChrA05 | 920330 | 924389 | 1089 | 120.0 | 4.86 |
| *Brara.H02675* | ChrA08 | 21367265 | 21368314 | 213 | 24.3 | 7.73 |
| *Brara.F03088* | ChrA06 | 24604295 | 24605790 | 414 | 46.5 | 6.58 |
| *Brara.F02119* | ChrA06 | 17891074 | 17892742 | 266 | 31.1 | 9.86 |
| *Brara.A01189* | ChrA01 | 6317686 | 6319763 | 460 | 53.3 | 8.89 |
| *Brara.I03509* | ChrA09 | 33039703 | 33041257 | 264 | 28.6 | 5.19 |
| *Brara.B03371* | ChrA02 | 28152628 | 28154548 | 482 | 54.8 | 5.78 |
| *Brara.B03788* | ChrA02 | 31245899 | 31247711 | 297 | 33.6 | 9.24 |
| *Brara.J01243* | ChrA10 | 11508683 | 11509430 | 152 | 17.52 | 9.00 |
| *Brara.B01527* | ChrA02 | 8466549 | 8468579 | 327 | 36.7 | 9.32 |
| *Brara.C04382* | ChrA03 | 23082391 | 23084307 | 220 | 23.6 | 4.88 |
| *Brara.C01447* | ChrA03 | 6953213 | 6955242 | 328 | 36.7 | 9.34 |
| *Brara.J02496* | ChrA10 | 17945717 | 17947218 | 307 | 34.7 | 9.23 |
| ***Oryza sativa*** | | | | | | |
| *LOC_Os09g35630* | Chr9 | 20494054 | 20496911 | 334 | 35.9 | 9.65 |
| *LOC_Os01g61500* | Chr1 | 35582263 | 35585727 | 262 | 28.7 | 5.63 |
| *LOC_Os05g43670* | Chr5 | 25390732 | 25392521 | 410 | 44.9 | 7.13 |
| *LOC_Os06g03640* | Chr6 | 1414221 | 1416618 | 339 | 36.4 | 9.71 |
| *LOC_Os08g43270* | Chr8 | 27357415 | 27359108 | 316 | 34.7 | 9.61 |
| *LOC_Os02g48780* | Chr2 | 29853571 | 29854458 | 213 | 23.0 | 5.99 |
| *LOC_Os02g15930* | Chr2 | 9021454 | 9023139 | 426 | 46.3 | 4.30 |
| *LOC_Os11g31060* | Chr11 | 18070199 | 18071815 | 455 | 49.7 | 4.70 |
| *LOC_Os02g01520* | Chr2 | 299744 | 302823 | 398 | 44.9 | 8.86 |
| *LOC_Os04g52890* | Chr4 | 31497254 | 31502769 | 272 | 29.1 | 9.14 |
| ***Glycine max*** | | | | | | |
| *Glyma.08G242700* | Chr08 | 20788215 | 20790091 | 452 | 50.0 | 8.57 |
| *Glyma.02G229300* | Chr02 | 41672433 | 41674966 | 268 | 29.5 | 6.42 |
| *Glyma.17G131600* | Chr17 | 10549941 | 10552445 | 274 | 31.0 | 9.42 |
| *Glyma.03G060900* | Chr03 | 9010786 | 9030397 | 540 | 59.8 | 5.03 |
| *Glyma.06G226500* | Chr06 | 32896778 | 32899625 | 101 | 11.2 | 5.32 |
| *Glyma.09G146700* | Chr09 | 36342769 | 36345462 | 353 | 39.4 | 9.49 |
| *Glyma.05G049600* | Chr05 | 4424533 | 4426856 | 275 | 31.3 | 9.58 |
| *Glyma.09G010100* | Chr09 | 788028 | 789958 | 189 | 21.6 | 6.21 |
| *Glyma.12G005300* | Chr12 | 415334 | 419034 | 398 | 45.7 | 9.47 |
| *Glyma.09G146500* | Chr09 | 36326696 | 36329401 | 340 | 37.9 | 9.44 |
| *Glyma.02G096400* | Chr02 | 8673826 | 8677424 | 338 | 39.1 | 5.17 |
| *Glyma.18G285100* | Chr18 | 56563807 | 56565132 | 377 | 43.4 | 4.90 |
| *Glyma.18G284900* | Chr18 | 56556144 | 56557078 | 303 | 35.0 | 5.21 |
| *Glyma.07G153700* | Chr07 | 18783691 | 18787806 | 254 | 28.4 | 5.16 |
| *Glyma.09G231200* | Chr09 | 45459818 | 45462673 | 402 | 46.3 | 9.42 |
| *Glyma.13G220500* | Chr13 | 33382281 | 33386506 | 253 | 28.3 | 9.11 |
| *Glyma.16G198500* | Chr16 | 35943480 | 35946032 | 307 | 34.3 | 9.74 |
| *Glyma.16G198600* | Chr16 | 35958085 | 35960833 | 354 | 39.6 | 9.24 |
| *Glyma.18G186200* | Chr18 | 44806639 | 4480954 | 679 | 75.2 | 4.42 |
| *Glyma.16G030000* | Chr16 | 2854583 | 2860239 | 1160 | 129.7 | 5.04 |
| *Glyma.01G123300* | Chr01 | 42526284 | 42535486 | 253 | 27.8 | 5.85 |
| *Glyma.01G121200* | Chr01 | 41625717 | 41627670 | 578 | 63.8 | 4.35 |
| *Glyma.18G264900* | Chr18 | 54986680 | 54988748 | 452 | 50.2 | 8.33 |
| *Glyma.10G238900* | Chr10 | 46734289 | 46735513 | 270 | 31.4 | 8.65 |
| *Glyma.15G091900* | Chr15 | 7077782 | 7081975 | 251 | 28.2 | 9.05 |
| *Glyma.15G114400* | Chr15 | 9018700 | 9021429 | 179 | 20.5 | 6.52 |
| *Glyma.07G061500* | Chr07 | 5454512 | 5460406 | 1162 | 130.7 | 4.97 |
| *Glyma.03G051600* | Chr03 | 6805437 | 6813768 | 250 | 27.5 | 5.24 |
| ***Vitis vinifera*** | | | | | | |
| *GSVIVT01000692001* | Chr19 | 15663551 | 15663916 | 121 | 14.0 | 8.98 |
| *GSVIVT01000691001* | Chr19 | 15646369 | 15646734 | 121 | 14.1 | 9.58 |
| *GSVIVT01030331001* | Chr8 | 8767790 | 8774501 | 223 | 24.9 | 5.93 |
| *GSVIVT01027028001* | Chr15 | 18496035 | 18501265 | 908 | 99.4 | 4.70 |
| *GSVIVT01021575001* | Chr10 | 7350497 | 7353093 | 219 | 24.5 | 9.00 |
| *GSVIVT01024693001* | Chr6 | 7674586 | 7678401 | 238 | 26.6 | 6.55 |
| *GSVIVT01011503001* | Chr14 | 30079465 | 30081266 | 156 | 17.4 | 10.62 |
| *GSVIVT01010619001* | Chr16 | 15947210 | 15949471 | 316 | 34.9 | 9.30 |
| *GSVIVT01018630001* | Chr19 | 11982872 | 11990729 | 259 | 29.2 | 9.59 |
| ***Zea mays*** | | | | | | |
| *GRMZM2G079956* | Chr1 | 197689846 | 197691677 | 358 | 37.1 | 10.55 |
| *GRMZM2G444845* | Chr6 | 62609089 | 62613582 | 316 | 33.6 | 9.79 |
| *GRMZM2G063162* | Chr5 | 139395266 | 139403105 | 1304 | 143.7 | 4.90 |
| *GRMZM2G120305* | Chr5 | 211846119 | 211848004 | 320 | 34.8 | 9.45 |
| *GRMZM2G116830* | Chr9 | 154199364 | 154201319 | 394 | 42.4 | 5.01 |
| *GRMZM2G080594* | Chr10 | 122836548 | 122837684 | 351 | 38.0 | 5.20 |
| *GRMZM2G075631* | Chr1 | 161636901 | 161638031 | 262 | 28.0 | 4.56 |
| *GRMZM2G035170* | Chr2 | 195285971 | 195287897 | 318 | 34.6 | 9.57 |
| *GRMZM2G361893* | Chr9 | 69369100 | 69370819 | 188 | 21.0 | 5.12 |
| *GRMZM2G097135* | Chr4 | 21342654 | 21344213 | 397 | 43.1 | 5.14 |
| *GRMZM2G400005* | Chr2 | 161644755 | 161646159 | 388 | 43.3 | 6.59 |
| *GRMZM2G017013* | Chr7 | 141613407 | 141615527 | 349 | 37.2 | 9.31 |
| *GRMZM2G383122* | Chr2 | 9132263 | 9137089 | 268 | 28.7 | 9.32 |
| *GRMZM2G110894* | Chr2 | 223483929 | 223485076 | 290 | 31.8 | 6.56 |
| *GRMZM2G029863* | Chr9 | 25081992 | 25084628 | 322 | 33.9 | 9.81 |
| *GRMZM2G011015* | Chr4 | 163885490 | 163886294 | 211 | 22.6 | 9.86 |
| *GRMZM5G812371* | Chr8 | 162179778 | 162181688 | 401 | 43.2 | 4.86 |
| *GRMZM2G046418* | Chr8 | 169242900 | 169246260 | 241 | 26.7 | 5.93 |
| *GRMZM2G382918* | Chr8 | 60333820 | 60334986 | 350 | 38.2 | 5.45 |
| *GRMZM2G382345* | Chr5 | 203687325 | 203688004 | 197 | 21.5 | 9.55 |
| *GRMZM2G157481* | Chr4 | 195189096 | 195190998 | 343 | 37.7 | 10.13 |
| *GRMZM2G018988* | Chr9 | 32531261 | 32533637 | 643 | 72.6 | 6.75 |
| *GRMZM2G014720* | Chr3 | 182952380 | 182955812 | 259 | 28.7 | 6.88 |
| *GRMZM2G410975* | Chr2 | 223476586 | 223477642 | 325 | 35.3 | 4.64 |
| *GRMZM2G472346* | Chr5 | 67505339 | 67511655 | 494 | 55.9 | 9.22 |

Chr; chromosome, aa; amino acid residue, pI; isoelectric point
